# Potassium humate supplementation improves photosynthesis and agronomic and yield traits of foxtail millet

**DOI:** 10.1038/s41598-024-57354-x

**Published:** 2024-04-25

**Authors:** Jie Shen, Xiaolu Xiao, Dandan Zhong, Huida Lian

**Affiliations:** 1 Department of Life Sciences, Changzhi University, Changzhi, 046011 China; 2 Oil Crops Research Institute, Chinese Academy of Agricultural Sciences, Wuhan, 430062 China; 3 College of Agronomy, Shanxi Agricultural University, Taigu, 030801 China

**Keywords:** Foxtail millet, Physiological attributes, Photosynthesis, Potassium humate, Yield, Plant sciences, Environmental sciences

## Abstract

Foxtail millet is a highly nutritious crop, which is widely cultivated in arid and semi-arid areas worldwide. Humic acid (HA), as a common plant growth regulator, is used as an organic fertilizer and feed additive in agricultural production. However, the impact of potassium humate KH on the photosynthetic rate and yield of foxtail millet has not yet been studied. We explored the effects of KH application on the morphology, photosynthetic ability, carbon and nitrogen metabolism, and yield of foxtail millet. A field experiment was performed using six concentrations of KH (0, 20, 40, 80, 160, and 320 kg ha^−1^) supplied foliarly at the booting stage in Zhangza 10 cultivar (a widely grown high-yield variety). The results showed that KH treatment increased growth, chlorophyll content (SPAD), photosynthetic rate (*P*n), transpiration rate (*T*r), and stomatal conductance (*G*s). In addition, soluble protein content, sugar content, and nitrate reductase activity increased in KH-treated plants. With increased KH concentration, the effects became more evident and the peak values of each factor were achieved at 80 kg ha^−1^. Photosynthetic rate showed significant correlation with SPAD, *T*r, *G*s, and soluble protein content, but was negatively correlated with intercellular CO_2_ concentration. Compared to that of the control, the yield of foxtail millet under the T2, T3, T4, and T5 (40, 80, 160, and 320 kg ha^−1^ of KH) treatments significantly increased by 6.0%, 12.7%, 10.5%, and 8.6%, respectively. Yield exhibited a significant positive correlation with *T*r, *P*n, and *G*s. Overall, KH enhances photosynthetic rate and yield of foxtail millet, therefore it may be conducive to stable millet production. These findings may provide a theoretical basis for the green and efficient production of millet fields.

## Introduction

Foxtail millet (*Setaria italica* L.) is one of the oldest domesticated crops in China^1^. As a multigrain crop, foxtail millet is highly cultivated in arid and semi-arid regions worldwide^2^. Due to its high stress tolerance, small genome, and short life cycle, foxtail millet has been utilized as a model for understanding the C_4_ photosynthesis, stress responses, and biofuel traits^3^. Previous research has revealed the greater nutritional value of foxtail millet compared to that of other major cereal grains^4^. Foxtail millet contains high quantities of resistant starch, free fatty acids, amino acids, vitamins, dietary fiber, and antioxidants^5^. In recent years, with increasing attention being given to healthy diets, the demand for foxtail millet has increased annually owing to its rich nutrient status^6^.

However, climate change poses a challenge to agricultural production and food security^7^. Although foxtail millet exhibits stress resistance, its growth is affected by abiotic stress at the critical stage, resulting in a significant reduction in yield^8^. Shanxi Province is the main foxtail millet-producing area in China with 200,000–230,000 ha of cultivated land, including for early-maturing spring sowing, mid-late-maturing spring sowing, and summer sowing^9^. As an arid and semi-arid region, Shanxi is the main area affected by agricultural drought disasters and mainly relies on rain-fed agriculture^10^. In addition, unconventional cultivation techniques (including the selection of plots and stubble, preparation and cultivation, sowing operation, fertilization management, and field management), diseases, and pest infestation affect grain yield^11^. Furthermore, in agricultural production, the use of large amount of fertilizer results in fertilizer wastage and environmental pollution, such as soil acidification and degradation and water eutrophication. In this context, the use of organic fertilizer has been avoided, thereby resulting in soil compaction and affecting crop yield^12^. Improving crop yield is a hot topic in modern agriculture.

Plant growth regulators (PGRs) are commonly used for agricultural purposes and effectively increase crop yield by improving the utilization of light energy to promote photosynthesis, enhance root activity, and increase stress resistance^13–16^. Humic acid (HA), a common PGR, is used as an organic fertilizer and feed additive in agricultural production^15–17^. HA can protect soil quality, enhance fertilizer consumption^16^, and promote crop yield and quality^19^ if applied in combination with inorganic fertilizers. Growth, yield, and the quality were enhanced in continuously cropped peanuts through improvement of the soil properties, including physicochemical properties, enzymatic functioning, and the microbial diversity^20^. HA promotes root growth by modulating the hormonal status like auxin functioning and production of NO^21^. The addition of HA to controlled-release fertilizers enhances yield and nitrogen uptake, thus improving nitrogen use efficiency in summer maize^22^. HA enhances the photosynthetic capacity of maize by improving the chlorophyll content (SPAD), photosynthetic rate (*P*n), transpiration rate (*T*r), and stomatal conductance (*G*s), thereby increasing the drought resistance of maize^23^. Potassium is an indispensable nutrient for plant growth and plays a crucial role in improving crop stress resistance, reducing pests and diseases, and improving crop quality and yield^24,25^. HA enhances potassium fertilizer use efficiency in tomatoes^26^. Furthermore, the combined application of potassium and HA can integrate their advantages, which is beneficial for the regulation of seed germination and plant growth and development^27^. Potassium humate (KH) is an efficient organic fertilizer that has positive effects on plant growth and productivity^28^, improving nutrient uptake^29^ and enhancing cotton productivity^30^. In addition, KH can increase growth parameters, including plant height, leaf area, and stem diameter. Moreover, the application of KH alleviates the inhibitory effects of soil salinity stress on bean plants^31^. The addition of KH to the leaf surface increases water productivity and results in a higher yield of corn^32^. KH significantly improves the yield, total biomass, and harvest index of tomatoes^33^. Furthermore, photosynthesis and quantum yield (Fv/Fm) are enhanced by KH addition^34^.

Millet yield decreases under low potassium stress conditions, which is mainly due to low utilization efficiency of light energy and reduced dry matter accumulation^35^. Enhancing the usage of light energy is the primary way to increase crop yields in production. However, there have been few reports on the impact of KH on the photosynthetic rate and yield of foxtail millet. Therefore, this study analyzed the effects of KH addition on the morphology, carbon and nitrogen metabolism, photosynthesis, and yield of foxtail millet using manipulative field experiments. The aims of the study included: (i) exploring the relationship between yield and yield-related components, (ii) revealing the significance of carbon and nitrogen metabolism and their relationship with yield, and (iii) determining the optimal KA application dosage. These findings can provide a theoretical basis for the green and efficient production of millet.

## Materials and methods

### Experimental design

**Figure 1:**
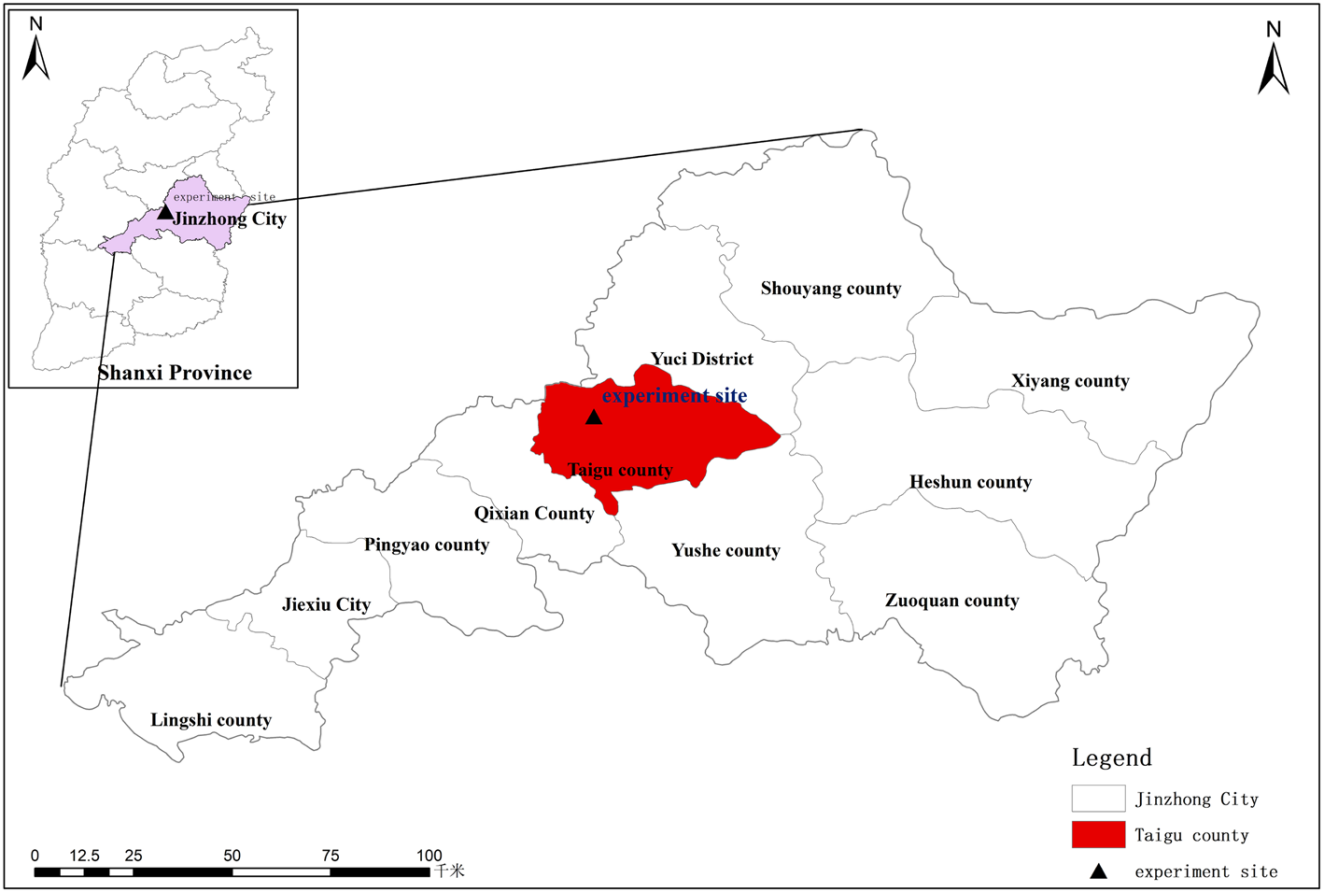
Experimental site location.

**Figure 2:**
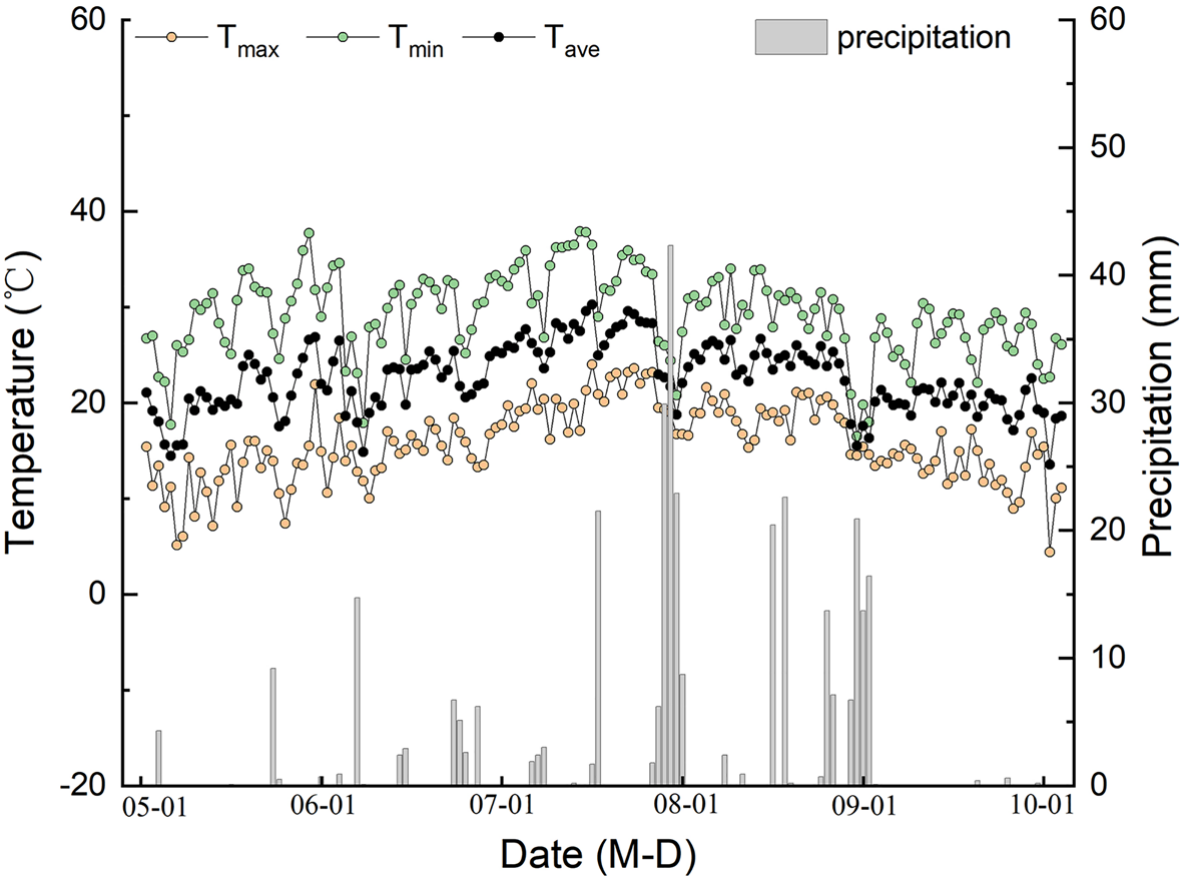
Mean daily maximum temperature (Tmax), mean daily minimum temperature (Tmin), average air temperature (Tave), and average daily precipitation during the Foxtail Millet-growing seasons in 2017.

The hybrid foxtail millet Zhangza 10 (Zhangjiakou City Academy of Agricultural Sciences, Hebei Province, China), a widely planted variety in the northern region of China, was grown in 2017 at the Shanxi Agricultural University Experimental Station (Shenfeng Village, Jinzhong City, Shanxi Province, China) (Fig. 1). The millet seeds were sown on May 24 and different doses of KH (organic humic acid ≥ 55%, fulvic acid ≥ 30%, potassium oxide ≥ 12%; Shanghai Bijiajia Biotechnology Co., LTD) were sprayed on the leaves of millet at booting stage (July 31). The concentrations of KH used were 0 kg ha^−1^ (CK), 20 kg ha^−1^(T1), 40 kg ha^−1^ (T2), 80 kg ha^−1^ (T3), 160 kg ha^−1^ (T4), and 320 kg ha^−1^ (T5). On clear and windless days, KH was applied evenly to the foliage using a handheld compression sprayer between 4 and 6 P.M. A completely randomized design was used for experiments with three replicates. There were 18 plots (3 × 3 = 9 m^2^) used. The precipitation and temperature data from May to September 2017 are shown in Fig. 2.

### Measurement of agronomic traits

After 10, 20, and 30 days of KH treatments, plant height, stem diameter, and leaf area were measured. Tape was used to record plant height and a vernier caliper was used for stem diameter measurement. Leaf area of the penultimate leaves was estimated using the formula:

$$ {\text{Leaf}}\;{\text{area}} = {\text{length }} \times {\text{width}} \times 0.{75} $$

### Measurement of chlorophyll content

Chlorophyll content was measured as the SPAD value using a SPAD-502 chlorophyll meter (Konica Minolta Holdings, Inc., Tokyo, Japan).

### Photosynthetic parameter estimation

At 10, 20, and 30 d after spraying KH, the photosynthetic parameters, including *P*n, *T*r, *G*s, and intercellular CO_2_ concentration (*C*i), were measured using a CI-340 photosynthesizer (CID Bio-Science, Inc., USA) between 9:00 and 11:00 on a clear day. Three penultimate millet leaves with the same growth pattern were measured under a light intensity of 900 ± 50 μmol m^−2^ s^−1^. The atmospheric CO_2_ concentration was 380 ± 50 μmol mol^−1^ and the air temperature were 30 ± 2℃.

### Measurement of the physiological traits of foxtail millet

Three plants with consistent growth were selected 10 d after spraying with KH, and the penultimate leaves of the millet were collected for the measurement of physiological traits.

To determine the soluble sugar content, first, 100 mg leaf tissue was extracted in 80% ethanol, and then the anthrone–sulfuric acid method described by Li^36^ was used.

Next, approximately 0.1 g of penultimate leaves, a small amount of quartz sand, and 2 mL of 50 mmol L^−1^ phosphate buffer (containing 0.1 mmol L^−1^ ethylenediaminetetraacetic acid and 1% polyvinylpyrrolidone) were mixed and ground in an ice bath. Then, the mixture was centrifuged at 15,000 × *g* for 15 min at 4℃. The supernatant was obtained and the amount of soluble protein was determined by Coomassie Brilliant Blue G-250 colorimetry^37^.

Subsequently, to assay the activity of nitrate reductase (NR), the method described by Srivastava^38^ was employed. Fresh leaves from each plot were incubated in 0.1 mol L^−1^ pH 7.5 KNO_3_ in the dark for 30 min. Thereafter, sulfonamide and α-naphthylamine reagent were added to the sample and left for 30 min. Then, the light absorption value required to convert NR activity was determined.

### Measurement of yield traits

At maturity, 20 plants from each treatment were selected to measure the yield parameters panicle diameter, panicle length, single panicle weight, panicle grain weight, spikelet number, and thousand grain weight. Seeds were air-dried at room temperature until they reached a constant weight. One square meter of foxtail millet was randomly harvested from each plot and the yield was measured.

### Comprehensive evaluation and statistical analysis

Microsoft Office Excel 2010 was used to analyze the data, and correlation and principal component analyses (PCA) were performed using IBM SPSS Statistics (Version 22.0; IBM SPSS Inc., USA) based on the method described by Jin^39^. Multiple comparisons were made using Duncan’s test at a significance level of *P* < 0.05, and data are presented as the mean of four replicates.

To determine the optimal application dosage of KH, the indexes for different concentrations of KH were analyzed comprehensively using the membership function value (MV) evaluation method in fuzzy mathematics. The fuzzy membership function is expressed as follows:

$$ {\text{V}}_{{{\text{ija}}}} = \left( {{\text{X}}_{{{\text{ij}}}} - {\text{X}}_{{{\text{jmin}}}} } \right) /\left( {{\text{X}}_{{{\text{jmax}}}} - {\text{X}}_{{{\text{jmin}}}} } \right) $$

$$ {\text{V}}_{{{\text{ijb}}}} = 1 - \left( {{\text{X}}_{{{\text{ij}}}} - {\text{X}}_{{{\text{jmin}}}} } \right) /\left( {{\text{X}}_{{{\text{jmax}}}} - {\text{X}}_{{{\text{jmin}}}} } \right) $$

where V_ija_ and V_ijb_ are the membership function values of index j treated by KH; V_ija_ and V_ijb_ represent the positive and negative correlations with the treatment, respectively; X_ij_ is the measured mean value of the index for different KH concentrations; and X_jmax_ and X_jmin_ are the maximum and minimum values of index j, respectively.

The final membership function value (MV) of comprehensive evaluation:

$$ {\text{MV}}_{{\text{i}}} = \frac{1}{{\text{n}}}\mathop \sum \limits_{{{\text{j}} = 1}}^{{\text{n}}} {\text{V}}_{{{\text{ij}}}} $$

The KH treatment concentration was assessed using the final membership function (MV). The higher the MV, the better the treatment concentration.

## Results

### Effects of KH application on yield parameters

**Figure 3:**
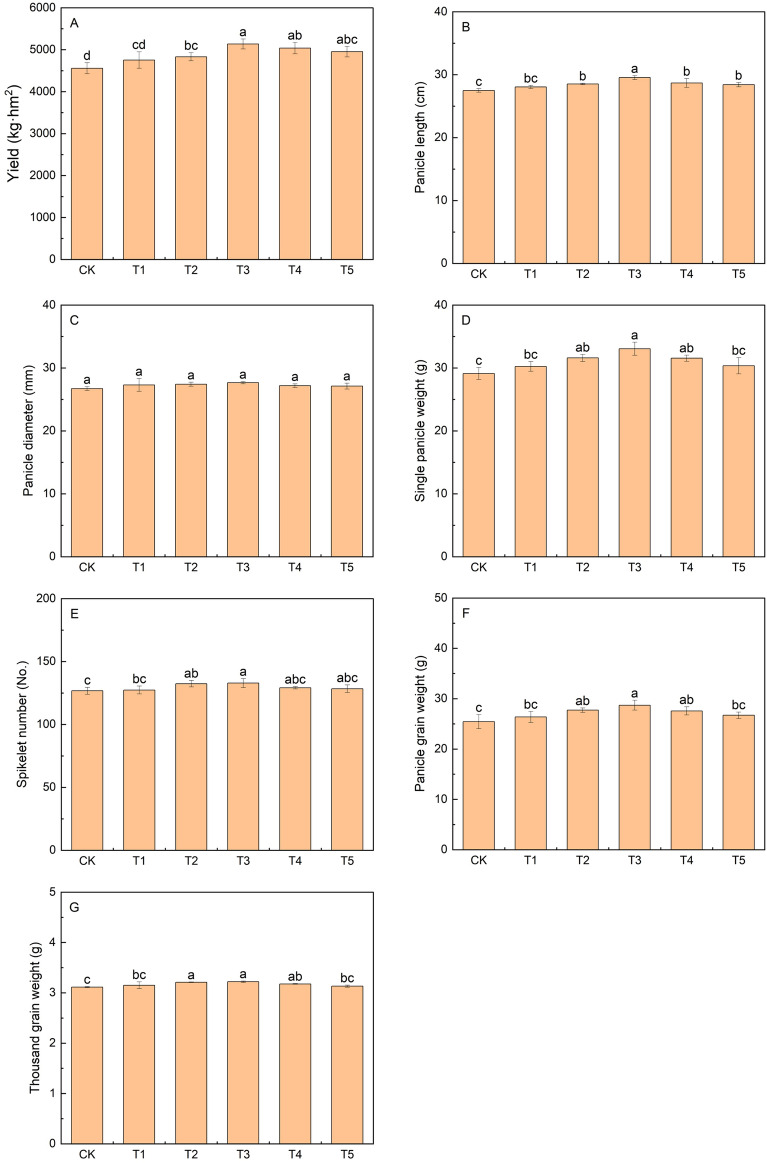
Effects of different potassium humate treatments on the yield (**A**), panicle length (**B**), panicle diameter (**C**), single panicle weight (**D**), spikelet number (**E**), panicle grain weight (**F**), and thousand gain weight (**G**) of foxtail millet. Different lowercase letters indicate significant differences at the *P* < 0.05. The concentrations of KH used were 0 kg ha^−1^ (CK), 20 kg ha^−1^(T1), 40 kg ha^−1^ (T2), 80 kg ha^−1^ (T3), 160 kg ha^−1^ (T4), and 320 kg ha^−1^ (T5).

The grain yield first increased and then decreased with increasing KH concentration and the highest yield was attained under the T3 treatment (Fig. 3). Compared to that of the CK group, crop yield increased by 6.0%, 12.7%, 10.5%, and 8.6% under the T2, T3, T4, and T5 treatments, respectively (*P* < 0.05). Panicle length, single panicle weight, spikelet number, panicle grain weight, and thousand grain weight exhibited an increase following KA application, with their maximum values observed in the T3 group, which were increased by 7.4%, 13.5%, 4.8%, 12.9%, and 3.5%, respectively, compared to that of the CK. Single panicle and panicle grain weight were significantly increased under T2, T3, and T4 compared to that of CK, and spikelet number was significantly different between T2 and T3.

### Effects of KH application on the agronomic traits of foxtail millet

**Figure 4:**
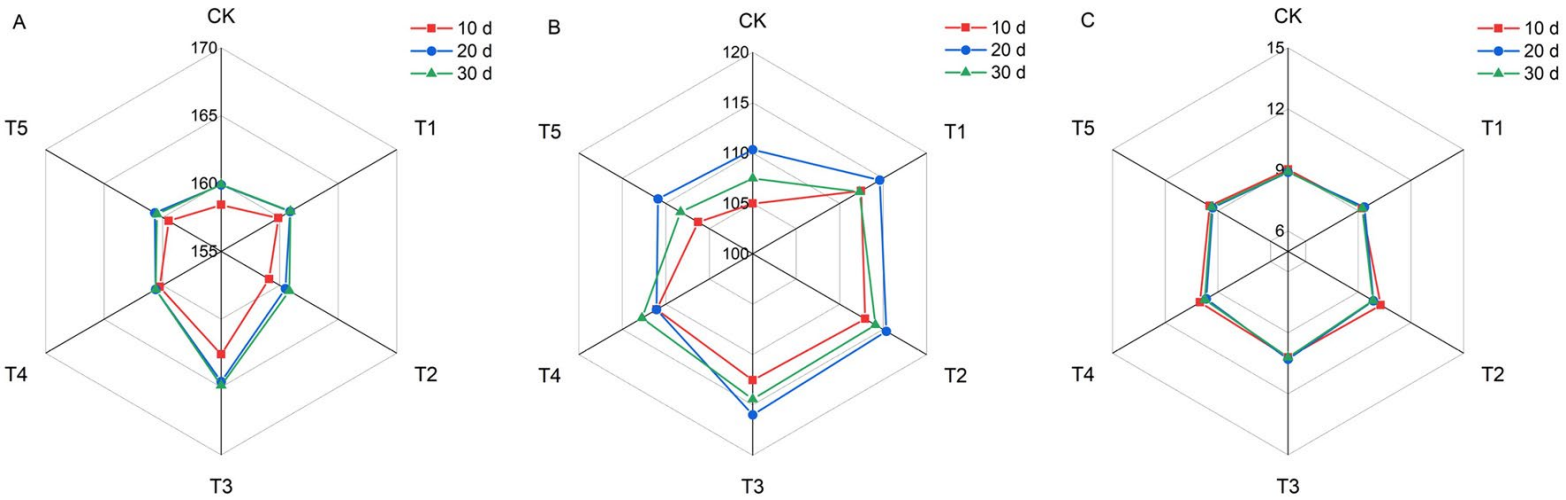
Effects of different potassium humate treatments on the agronomic traits of foxtail millet. Plant height (**A**), stem diameter (**B**) and leaf area (**C**). The concentrations of KH used were 0 kg ha^−1^ (CK), 20 kg ha^−1^(T1), 40 kg ha^−1^ (T2), 80 kg ha^−1^ (T3), 160 kg ha^−1^ (T4), and 320 kg ha^−1^ (T5).

Different KH treatments resulted in considerable differences in the plant height and stem diameter of millet in the same growth stage, and plant height and stem diameter exhibited an increase with increasing KH concentration (Fig. 4). At 10, 20, and 30 d, the maximum plant height was achieved under the T3 treatment, which significantly was increased by 2.6%, 2.9%, and 3.1%, respectively, compared with that of the CK group. The maximum stem diameter at 20 and 30 d after KH addition was also attained under the T3 treatment, which was significantly increased by 15.9% and 14.6%, respectively compared with that of CK. There were no significant differences in millet leaf area between the different KH concentrations.

### Effects of KH application on the SPAD values of foxtail millet

**Figure 5:**
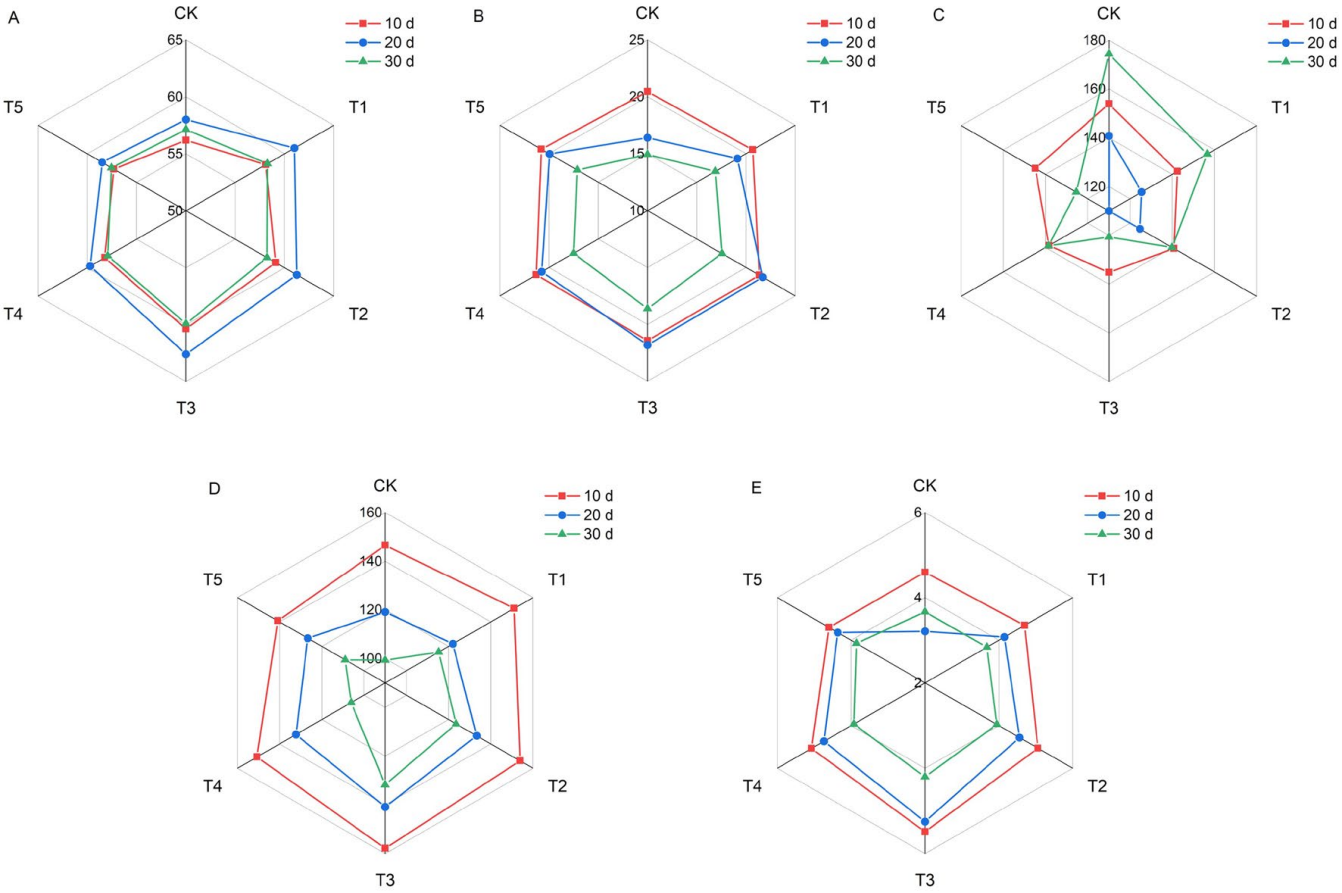
Effects of different potassium humate treatments on the SPAD value and photosynthetic gas exchange of foxtail millet: SPAD value (**A**), photosynthetic rate (**B**), intercellular CO_2_ concentration (**C**), stomatal conductance (**D**), and transpiration rate (**E**). The concentrations of KH used were 0 kg ha^−1^ (CK), 20 kg ha^−1^(T1), 40 kg ha^−1^ (T2), 80 kg ha^−1^ (T3), 160 kg ha^−1^ (T4), and 320 kg ha^−1^ (T5).

KH had a positive effect on the SPAD values of millet (Fig. 5A). With increasing KH concentration, the SPAD values first increased, peaked under T3, and then decreased. SPAD values were significantly increased by 7.4%, 7.9%, and 4.9% at 10, 20, and 30 d under T3, respectively, compared with those of CK.

### Effects of KH application on photosynthetic gas exchange in foxtail millet

Application of KH could affected photosynthetic gas exchange in millet (Fig. 5B–E). However, the changes in *P*n, *T*r, *G*s, and *C*i in response to KH treatment were inconsistent. Treatment with KH increased the values of *P*n, *T*r, and *G*s but reduced *C*i in millet leaves. The T3 treatment had the greatest effect on *Pn*, *T*r, *G*s, and *C*i, and significantly increased *P*n by 33.0% and 24.7% at 20 and 30 d, respectively. Additionally, *T*r was significantly increased by 19.4% and 63.6% on 10 and 20 d, respectively, compared with that of the CK group. Meanwhile, the *G*s significantly increased by 7.7%, 18.3%, and 32.7%, whereas *C*i significantly decreased by 12.1%, 31.6%, and 30.8% at 10, 20, and 30 d, respectively, compared with those of CK.

### Effects of KH application on the physiological characteristics of foxtail millet

**Figure 6:**
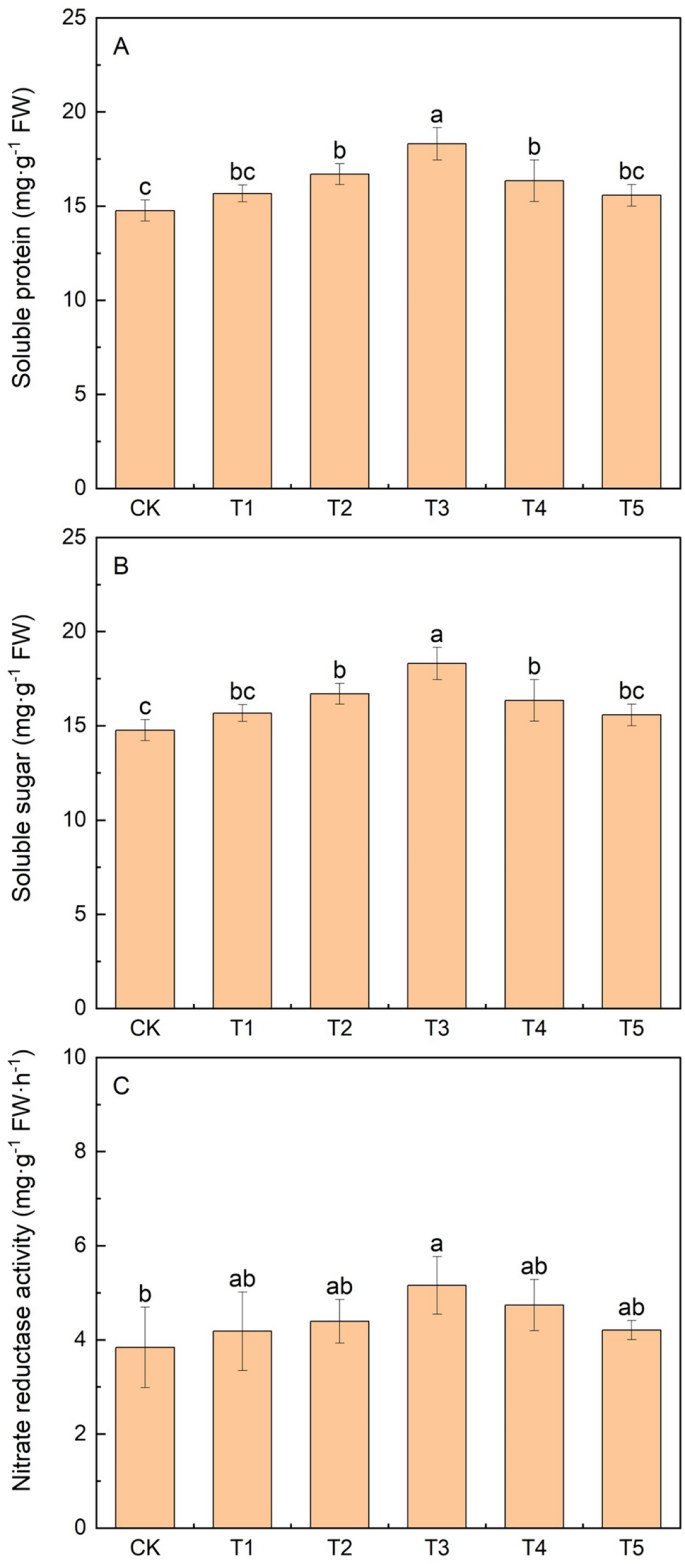
Effects of different potassium humate treatments on the physiological characteristics of foxtail millet: Soluble protein content (**A**), soluble sugar content (**B**), and nitrate reductase activity (**C**). Different lowercase letters indicate significant differences at *P* < 0.05. The concentrations of KH used were 0 kg ha^−1^ (CK), 20 kg ha^−1^(T1), 40 kg ha^−1^ (T2), 80 kg ha^−1^ (T3), 160 kg ha^−1^ (T4), and 320 kg ha^−1^ (T5).

Ten days after KH application during the booting stage, both the soluble protein and soluble sugar contents in leaves increased. The soluble protein content first increased and then decreased with increasing KH concentration, whereas the soluble sugar content increased gradually. Under T3, soluble protein content increased by 23.9% compared with that of the CK (Fig. 6). The soluble sugar content was the highest under T5, which was significantly increased by 19.1% compared with that of CK. The activity of nitrate reductase was significantly increased by 34.4% under T3 compared with that of CK, while that exhibited under other treatments showed no significant difference.

### Membership function, principal component, and correlation analysis of related traits under KH treatment

The MV method can be used as a comprehensive method to determine the optimal application dosage of KH for foxtail millet according to the yield and morphological, photosynthetic, and physiological indices under different KH dosage levels. The MV was 0.34, 0.64, 0.97, 0.68, and 0.49 under T1–T5 (20, 40, 80, 160, and 320 kg ha^−1^ KH, respectively) (Table 1 and S1). MV was higher under T3 than under the other treatments. Therefore, 80 kg ha^−1^ KH was used as the optimal concentration based on the MV method.

**Table 1:** Evaluation of different potassium humate (KH) concentrations using the membership function value (MV) method.

Treatment	MV	Rank
CK	0.01	6
T1	0.34	5
T2	0.64	3
T3	0.97	1
T4	0.68	2
T5	0.49	4

**Table 2:** Correlation analysis of the yield, yield-related components, agronomic traits, and physiological attributes in foxtail millet: plant height (PH), stem diameter (SD), leaf area (LA), SPAD, soluble protein (SP), soluble sugar (SS), nitrate reductase (NR), panicle length (PL), panicle diameter (PD), single panicle weight (SPW), spikelet number (SN), panicle grain weight (PGW), thousand gain weight (TGW), and grain yield (GY). Different lowercase letters indicate significant differences at *P* < 0.05.

	PH	SD	LA	SPAD	*P*n	*T*r	*G*s	*C*i	SP	SU	NR	PL	PD	SPW	SN	PGW	TGW
PH	1																
SD	0.86	1															
LA	0.42	0.16	1														
SPAD	0.89*	0.92*	0.33	1													
*P*n	0.86*	0.82*	0.66	0.89*	1												
*T*r	0.90*	0.80	0.69	0.89*	0.99*	1											
*G*s	0.94*	0.72	0.61	0.85*	0.85*	0.91*	1										
*C*i	-0.98*	-0.83*	-0.59	-0.89*	-0.93*	-0.96*	-0.97*	1									
SP	0.90*	0.64	0.65	0.79	0.88*	0.92*	0.90*	-0.93*	1								
SU	0.34	0.22	0.76	0.13	0.55	0.53	0.35	-0.46	0.47	1							
NR	0.58	0.74	0.58	0.67	0.87*	0.80	0.54	-0.69	0.55	0.67	1						
PL	0.89*	0.76	0.67	0.92*	0.96*	0.98*	0.94*	-0.95*	0.91*	0.39	0.71	1					
PD	0.97*	0.95*	0.34	0.92*	0.87*	0.89*	0.88*	-0.95*	0.82*	0.35	0.68	0.86*	1				
SPW	0.82*	0.54	0.76	0.70	0.88*	0.91*	0.84*	-0.87*	0.98*	0.60	0.60	0.89*	0.73	1			
SN	0.70	0.53	0.49	0.58	0.77	0.75	0.57	-0.71	0.85*	0.60	0.57	0.67	0.66	0.88*	1		
PGW	0.94*	0.77	0.40	0.71	0.75	0.79	0.84*	-0.90*	0.83*	0.51	0.53	0.73	0.92*	0.76	0.72	1	
TGW	0.97*	0.89*	0.50	0.95*	0.95*	0.97*	0.93*	-0.99*	0.91*	0.39	0.72	0.95*	0.97*	0.85*	0.72	0.87*	1
GY	0.86*	0.81	0.55	0.93*	0.97*	0.96*	0.83*	-0.90*	0.90*	0.38	0.76	0.96*	0.86*	0.88*	0.80	0.71	0.94*

**Figure 7:**
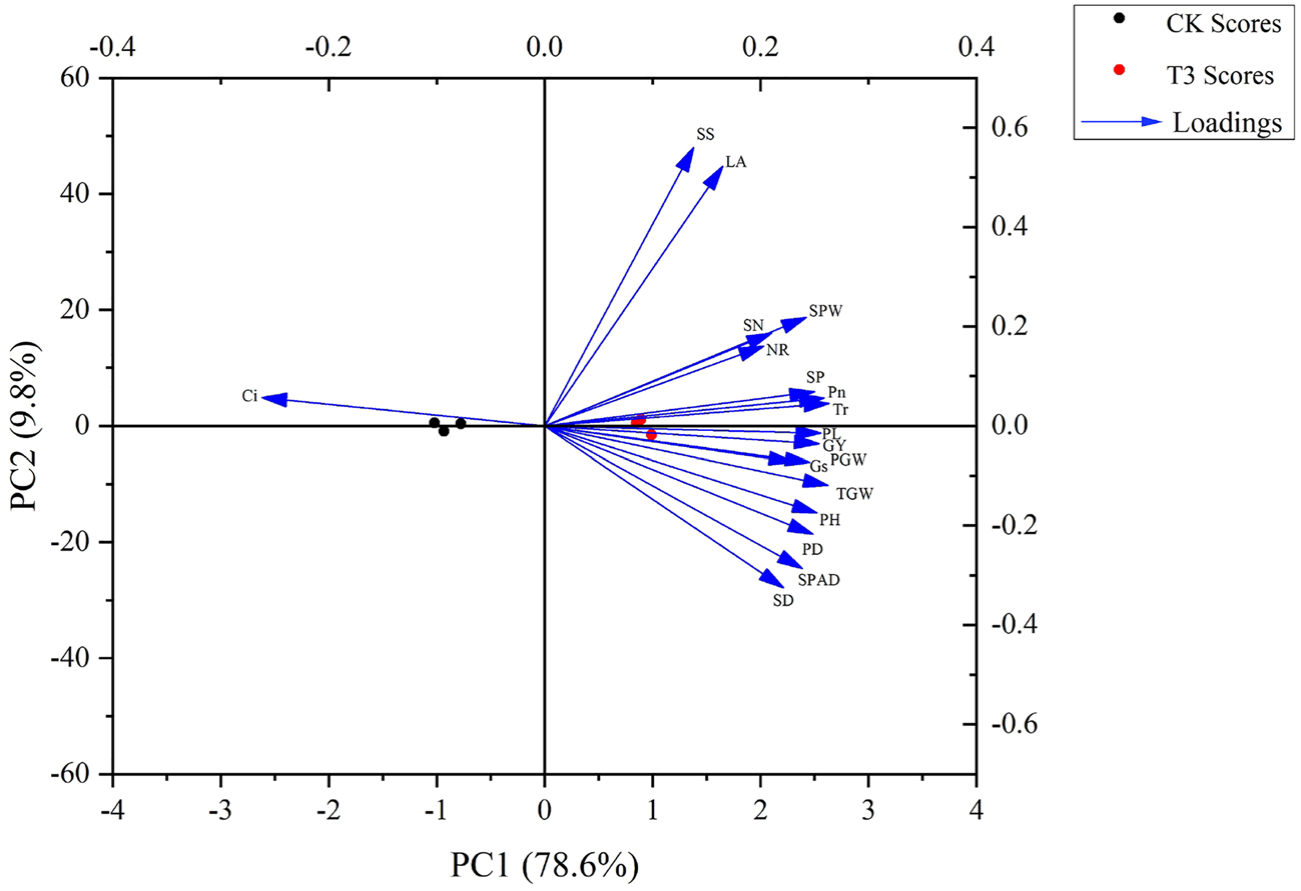
PCA of the yield, yield-related components, agronomic traits, and physiology attributes in foxtail millet: Plant height (PH), stem diameter (SD), leaf area (LA), SPAD, soluble protein (SP), soluble sugar (SS), nitrate reductase (NR), panicle length (PL), panicle diameter (PD), single panicle weight (SPW), spikelet number (SN), panicle grain weight (PGW), thousand gain weight (TGW), and grain yield (GY).

Principal component analysis (PCA) and correlation analysis were used to evaluate the degree of contribution of each index to the optimal treatment concentration (T3). To explain the genotype differences and differences in the 21 indices between the treatments, two principal components (PC 1 and PC 2) were utilized and the cumulative contribution rate was 88.4% (Fig. 7 and Table S2). PC 1 represented 78.6% of the variability, whilst PC 2 contributed 9.8%. As shown in Fig. 7, PC1 was negatively correlated with *C*i but positively correlated with the other indices. Moreover, PC2 was positively correlated with soluble sugar content, leaf area, spikelet number, single panicle weight, nitrate reductase, soluble protein content, photosynthetic rate, and transpiration rate but negatively correlated with the other indices. Therefore, the two main components showed the comprehensive effects of different KH treatments on foxtail millet growth. Figure 7 shows that plant height, *P*n, *T*r, panicle length, thousand grain weight, and grain yield, the loadings of other indexes, were larger in PC1, suggesting that the larger the horizontal axis value, the more conducive to the accumulation of dry matter and the growth and development of foxtail millet.

Yield showed a significant strong positive correlation with plant height, SPAD, *P*n, *T*r, *G*s, soluble protein content, panicle length, panicle diameter, single panicle weight, and thousand gain weight (*P* < 0.05, Table 2). In addition, *P*n was positively correlated with SPAD, *T*r, *G*s, soluble protein content, and nitrate reductase but was negatively correlated with *C*i, indicating that the enhancement of yield by KH was related to photosynthesis. This confirms that the increase in *P*n due to KH was related to its influence on the stomatal parameters as well as non-stomatal parameters.

## Discussion

### Application of KH increased yield in foxtail millet

KH is a promising natural resource that can be utilized as an alternative to fertilizers to increase crop production^40^. Studies have shown that KH significantly improves the yield of rice^40^, *Vicia faba*^28^, potato^41^, and wheat^42^. The present study showed that an appropriate concentration of KH increased panicle length, panicle weight, spikelet number, panicle grain weight, and thousand grain weight and significantly improved the yield. In particular, when the concentration was 80 kg ha^−1^, the yield and yield-associated attributes were greatly increased (Fig. 3). Correlation analysis of plant characteristics at 80 kg ha^−1^ showed that the enhancement of yield by KH was related to plant height, SPAD, *P*n, *T*r, *G*s, soluble protein content, panicle length, panicle diameter, single panicle weight, and thousand gain weight (Table 2).

### Application of KH promoted the growth of and photosynthesis in foxtail millet

The booting stage of millet is a period of vegetative and reproductive growth. Fertilization at this stage is beneficial for the accumulation of photosynthates and panicle differentiation, which determine the yield^40^. Application of KH significantly improved the growth traits of millet compared with those of CK, particularly the addition of 80 kg ha^−1^, which significantly increased millet plant height and stem diameter (Fig. 4). Optimal KH concentrations enhanced the growth and development of *Vicia faba*^28^. This may be related to increased IAA content, which can affect cell elongation and promote growth traits^23^. Here, SPAD values significantly increased in plants treated with 80 kg ha^−1^ KH (Fig. 5). Potassium humate promotes hormone production, stimulates root growth, and enhances the uptake of minerals, such as Fe^43^, therefore mediating increased synthesis of chlorophyll. In this study, correlation analysis revealed that SPAD values in leaves were positively correlated with *P*n, indicating improved photosynthesis in millet leaves (Table 2). The application of humic acid and potassium fertilizers improves leaf photosynthesis by altering the gas exchange parameters^23,44^. In the present study, KH application increased *P*n, *T*r, and *G*s but reduced *C*i, which suggests the potential of mesophyll cells for CO_2_ assimilation increased. Furthermore, the increase in *P*n accompanied by an increase in the SPAD value and decrease in *C*i indicates that both chlorophyll and the non-stomatal attributes influence the *P*n, thus improving the photosynthesis rate^13,45^. Correlation analysis showed that *P*n and *G*s were significantly positively correlated, indicating that enhanced photosynthesis in millet due to KH treatment is related to the influence of KH influence on stomatal factors. These findings are in line with those of Feng^13^. Hence, a suitable KH concentration can enhance millet photosynthesis by improving stomatal and the stomatal photosynthetic attributes (Table 2).

### Application of KH changed the physiological properties of foxtail millet

Plant growth and development is considerably influenced by the availability of carbohydrates for photosynthesis (source activity) and their utilization for growth (sink activity)^46^. Soluble sugars are the main carbohydrate components that are inter-converted and reused^47^. In the current study, treatment of 320 kg ha^−1^ KH maximally increased the soluble sugar content of foxtail millet, thereby increasing the yield. Soluble protein content directly affects plant photosynthesis. A previous study suggested that 50% of the soluble proteins in plant leaves were RuBP carboxylase, which is a key enzyme in photosynthesis^48^. Our results showed that protein content significantly increased following the addition of 40–160 kg ha^−1^ KH compared to that in the control, indicating increased soluble protein synthesis due to KH application (Fig. 6). Nitrate reductase is a key enzyme in the nitrogen assimilation pathway^49^. Lu^50^ showed that nitrate reductase is highly promoted by an increase in HA. The present study showed that a suitable KH concentration can significantly improve the activity of nitrate reductase in millet leaves. Increased nitrate reductase activity affects the ability of plants to absorb external nitrogen, thereby contributing to increasing the crop yield^51^.

### Comprehensive evaluation of the effects of KH on foxtail millet

The MV method is widely used to screen treatment concentrations for crop growth and resistance for abiotic stress^52,53^. Our study investigated the efficacy of different KH concentrations using MV and the optimal KH concentrations required for promoting the growth and yield of foxtail millet were determined. The final MV results can be utilized to obtain the optimal concentration treatment; the larger the MV, the better the effect of KH on foxtail millet. A concentration of 80 kg ha^−1^ resulted in a higher MV value (Table 1 and S1) and had a greater effect on Zhangza 10 than those of the other treatments. Therefore, 80 kg ha^−1^ can be used as the optimal KH concentration during the booting stage of foxtail millet.

## Conclusions

Application of KH at the booting stage promoted the growth of Zhangza 10 cultivar by increasing the chlorophyll content (SPAD value), *P*n, *T*r, *G*s, protein content, soluble sugar content, and the activity of nitrate reductase. Potassium humate tend to increased maximally at T3 and thereafter showed a declining trend. Yield showed a significant positive correlation with *T*r, *P*n, and *G*s. Further, the KH-induced enhancement of the photosynthetic attributes and yield was conducive to millet production stability. The optimal potassium application concentration for Zhangza 10 is 80 kg ha^−1^.

### Supplementary Information

Supplementary Tables.

## Data Availability

This manuscript includes all data generated or analyzed during this study. Other necessary data of this study are available with the corresponding author on reasonable request.
