# Supplementary material for: Potassium humate supplementation improves photosynthesis and agronomic and yield traits of foxtail millet
Source: Sci Rep. 2024 Apr 25;14:9508. doi: 10.1038/s41598-024-57354-x (PMC11045805; doi:10.1038/s41598-024-57354-x)
Supplement: Supplementary file 1 — Supplementary Tables. [file 41598_2024_57354_MOESM1_ESM.docx]

**Supplementary table legend**

**Table S1. The comprehensive evaluation value of different Potassium humate treatments.**

| **Treatment** | **Grain**  **Yeild** | **Panicle length** | **Panicle diameter** | **Single panicle weight** | **Spikelet number** | **Panicle grain weight** | **Thousand grain weight** | **Plant height** | **Stem diameter** | **Leaf area** | **SPAD** | ***P*n** | **Tr** | ***G*s** | ***C*i** | **Soluble protein** | **Soluble sugar** | **Nitrate reductase activity** | **MFV** | **Rank** |
| --- | --- | --- | --- | --- | --- | --- | --- | --- | --- | --- | --- | --- | --- | --- | --- | --- | --- | --- | --- | --- |
| **CK** | **0.00** | **0.00** | **0.00** | **0.00** | **0.00** | **0.00** | **0.00** | **0.00** | **0.00** | **0.00** | **0.00** | **0.00** | **0.00** | **0.11** | **0.00** | **0.00** | **0.00** | **0.00** | **0.01** | **6** |
| **T1** | **0.34** | **0.27** | **0.62** | **0.29** | **0.11** | **0.28** | **0.35** | **0.25** | **0.24** | **0.80** | **0.51** | **0.43** | **0.20** | **0.41** | **0.43** | **0.25** | **0.04** | **0.26** | **0.34** | **5** |
| **T2** | **0.76** | **0.50** | **0.75** | **0.63** | **0.91** | **0.70** | **0.90** | **0.15** | **0.80** | **0.95** | **0.60** | **0.87** | **0.57** | **0.72** | **0.57** | **0.55** | **0.16** | **0.42** | **0.64** | **3** |
| **T3** | **1.00** | **1.00** | **1.00** | **1.00** | **1.00** | **1.00** | **1.00** | **1.00** | **0.98** | **0.98** | **1.00** | **1.00** | **1.00** | **1.00** | **1.00** | **1.00** | **0.46** | **1.00** | **0.97** | **1** |
| **T4** | **0.83** | **0.58** | **0.50** | **0.62** | **0.40** | **0.65** | **0.59** | **0.24** | **0.66** | **2.51** | **0.38** | **0.79** | **0.59** | **0.47** | **0.78** | **0.45** | **0.49** | **0.68** | **0.68** | **2** |
| **T5** | **0.68** | **0.45** | **0.42** | **0.32** | **0.27** | **0.39** | **0.17** | **0.18** | **0.33** | **2.08** | **0.18** | **0.53** | **0.31** | **0.21** | **0.70** | **0.23** | **1.00** | **0.28** | **0.49** | **4** |

**Table S2. Principal component analysis of growth and physiological parameters under Potassium humate treatment of foxtail millet.**

|  | PC1 | PC2 |
| --- | --- | --- |
| Plant height | 0.25197 | -0.17466 |
| Stem diameter | 0.221 | -0.32504 |
| Leaf area | 0.1647 | 0.52262 |
| SPAD | 0.23853 | -0.28568 |
| Pn | 0.25879 | 0.05567 |
| Tr | 0.26294 | 0.04512 |
| Gs | 0.24538 | -0.07321 |
| Ci | -0.26165 | 0.05682 |
| Soluble protein | 0.24981 | 0.06839 |
| Soluble sugar | 0.13802 | 0.56061 |
| Nitrate reductase activity | 0.20267 | 0.16071 |
| Panicle length | 0.25567 | -0.01468 |
| Panicle diameter | 0.24841 | -0.21722 |
| Single panicle weight | 0.24209 | 0.21865 |
| Spikelet number | 0.21007 | 0.18637 |
| Panicle grain weight | 0.23053 | -0.07207 |
| Thousand grain weight | 0.26244 | -0.11917 |
| Grain yield | 0.254 | -0.03559 |
